# Retinal changes and cardiac biomarker assessment in relation to chronic kidney disease: a single centre study

**DOI:** 10.1186/s12882-023-03386-w

**Published:** 2023-11-13

**Authors:** Ruslinda Mustafar, Khairun Amalin Mohd Hishamuddin, Rozita Mohd, Lydia Kamaruzaman, Wan Haslina Wan Abdul Halim, Yong Meng Hsien, Tan Kuan Sze, Wan Mimi Diyana Wan Zaki, Aziah Ali, Arbaiyah Bain

**Affiliations:** 1 Department of Medicine, Faculty of Medicine, Universiti Kebangsaan Malaysia, Kuala Lumpur, Malaysia; 2 Department of Ophthalmology, Faculty of Medicine, Universiti Kebangsaan Malaysia, Kuala Lumpur, Malaysia; 3 Department of Electrical, Electronic and Systems Engineering, Faculty of Engineering and Built Environment, Universiti Kebangsaan Malaysia, Bangi, Malaysia; 4 Faculty of Computing and Informatics, Multimedia University, Cyberjaya, Malaysia

**Keywords:** Retinal vessel calibre, Retinal vessel tortuosity, Macular volume, Cardiac biomarker, Chronic kidney disease, Cardiovascular risk

## Abstract

**Background:**

The prevalence of chronic kidney disease (CKD) is rising in Malaysia. Early detection is necessary to prevent disease progression, especially in terms of cardiovascular (CV) risk, the main cause of death in end-stage renal disease (ESRD). Retinal changes have proven to be a good predictor of CKD whereas cardiac biomarkers are useful in cardiovascular risk stratification. We aimed to demonstrate the correlation between retinal changes and cardiac biomarkers with CKD.

**Methods:**

This single-centre cross-sectional study was conducted among patients with CKD stages 3, 4, and 5 (not on dialysis) from the Nephrology Clinic, Universiti Kebangsaan Malaysia Medical Centre. A total of 84 patients were recruited with an even distribution across all three stages. They underwent fundus photography where images were analysed for vessel calibre (central retinal venular equivalent (CRVE), central retinal arterial equivalent (CRAE), and tortuosity indices. Optical coherence tomography was used to measure macular volume. Blood samples were sent for laboratory measurement of high-sensitivity C-reactive protein (hs-CRP) and asymmetric dimethylarginine (ADMA). These parameters were analysed in relation to CKD.

**Results:**

The mean age was 58.8 ± 11.7 years, with 52.4% male and 47.6% female patients. Among them, 64.3% were diabetics. Retinal vessel tortuosity (*r* = -0.220, *p*-value = 0.044) had a negative correlation with the estimated glomerular filtration rate (eGFR). CRVE showed a positive correlation with proteinuria (*r* = 0.342, *p* = 0.001) but negative correlation with eGFR (*r* = -0.236, *p* = 0.031). Hs-CRP positively correlated with proteinuria (*r* = 0.313, *p* = 0.04) and negatively correlated with eGFR (*r* = -0.370, *p* = 0.001). Diabetic patients had a higher CRVE compared to non-diabetic patients (*p* = 0.02). History of ischaemic heart disease was associated with a smaller macula volume (*p* = 0.038). Male gender (*r*^2^ = 0.066, *p* = 0.031) and HbA1c had a positive influence (*r*^2^ = 0.066, *p* = 0.047) on retinal vessel tortuosity. There was a positive influence of age (*r*^2^ = 0.183, *p* = 0.012) and hs-CRP (*r*^2^ = 0.183, *p* = 0.045) on CRVE. As for macula volume, it negatively correlated with diabetes (*r*^2^ = 0.015, *p* = 0.040) and positively correlated with smoking (*r*^2^ = 0.015, *p* = 0.012).

**Conclusion:**

Our study showed that eGFR value affects retinal vessel tortuosity, CRVE and hs-CRP. These parameters bear potential to be used as non-invasive tools in assessing CKD. However, only macula volume may be associated with CVD risk among the CKD population.

## Background

The optimal management approach of chronic kidney disease (CKD) should be focused on preventive strategy as there is currently no cure for CKD. Early detection and intervention are required to prevent the progression to end-stage renal disease (ESRD). The prevalence of CKD in Malaysia has drastically risen from 9.07% in 2011 to 15.48% in 2018, likely attributable to the concurrent rise in the prevalence of risk factors such as diabetes mellitus (DM), hypertension, obesity, and increasing age [1].

The recent use of non-invasive methods in detecting early retinal changes such as retinal vessel tortuosity and calibre as well as macula volume has been shown to enhance CKD identification and risk prediction in earlier stages as these retinal changes precede changes such as diabetic retinopathy [2]. With earlier detection, more proactive interventions can be implemented without having to perform additional risky procedures such as renal biopsy, especially in cases whereby the aetiology of CKD is still doubtful.

To date, many studies have indicated that retinal changes are good predictors of CKD outcomes [2, 3]. One of the possible reasons is the fact that the kidney and the retina share several similarities. Anatomically, they share the same developmental pathway. Physiologically, the renin–angiotensin–aldosterone system is present in both these organs. Thus, factors leading to CKD such as atherosclerosis, vascular remodelling, endothelial dysfunction, and oxidative stress can also predispose to many retinal diseases [4].

Additionally, previous studies have shown that increased retinal venular diameter (measured by means of central retinal venular equivalent measurement, CRVE) was associated with an increased risk of CKD [2, 5]. However, this was not replicated in other studies [6–8]. In contrast, other publications revealed that smaller retinal arterioles (measured by central retinal arteriolar equivalent, CRAE) were associated with an increased risk of CKD [3, 5, 7, 8]. Other changes such as reduced macula volume [9] and increased tortuosity of retinal vessels [10] were also found in those with CKD.

In addition, the assessment of cardiovascular risk is vital in the early stage of CKD diagnosis as CVD is the leading cause of death in ESRD. Previous studies reported that patients with CKD were found to be in a chronic low-grade pro-inflammatory state which would promote atherosclerosis, thus leading to the conclusion that CKD in itself is a risk factor for CVD [11]. Cardiac biomarkers such as high-sensitivity C-reactive protein (hs-CRP) and asymmetric dimethylarginine (ADMA) have also been shown to be elevated in those with cardiovascular risk [12, 13]. Hs-CRP is a biomarker of inflammation which plays a key role in atherosclerosis. Previous studies have consistently proven that CRP levels independently predict the first episode of cardiovascular event across all Framingham risk groups [14–16]. ADMA, on the other hand, is a competitive inhibitor for endothelial nitric oxide synthase, which leads to endothelial dysfunction, and consequently circulating ADMA levels adds prognostic value towards cardiovascular risk [17, 18]. Therefore, these biomarkers were good predictors of CVD, even among CKD patients [11, 19, 20]. In short, these novel non-invasive tools for assessment of CKD and cardiovascular risk represent the future of risk prediction methods. Therefore, we embarked on this study to determine the correlation between retinal changes and cardiac biomarkers with CKD stages.

## Methodology

### Study population

This was a single-centre cross-sectional study involving patients above 18 years old who attended the Nephrology Clinic of Universiti Kebangsaan Malaysia Medical Centre (UKMMC) between October 2019 and January 2021. They must have an eGFR of < 60 ml/min/1.73m^2^, stable serum creatinine for three months, and HbA1c of < 10% if diabetic. Those with active malignancy, infection or vasculitis, on dialysis, pregnant, and history of renal transplant or retinal photocoagulation therapy were excluded. Written consent was obtained before the blood sample was taken for cardiac biomarkers. All participants were then sent to the Ophthalmology Department for fundus photography and Optical Coherence Tomography (OCT). Ethical approval was obtained from the Research and Ethics Committee UKM (FF-2019–425).

eGFR was calculated based on the Modification of Diet in Renal Disease (MDRD) Eq. [21]. Four variables were used whereby Glomerular filtration rate (GFR) (mL per minute per 1.73m^2^) = 175 × SerumCr-1.154 × age-0.203 × 1.212 (if patient is black) × 0.742 (if female).

CKD stage was classified as eGFR: stage 1 (> 90 ml/min/1.73m^2^), stage 2 (60 to 89 ml/min/1.73m^2^), stage 3a (45 to 59 ml/min/1.73m^2^), stage 3b (30 to 44 ml/min/1.73m^2^), stage 4 (15 to 29 ml/min/1.73m^2^) and stage 5 (less than 15 ml/min/1.73m^2^).

### Sample size

Based on the sample size calculation using a power of 80%, alpha-value of 0.05, and effect size of 0.3, a total of 82 patients was needed. The effect size of 0.3 indicated a moderate correlation and it was based on a previous study [9].

### Demographic data and laboratory parameters assessment

All participants underwent a face-to-face interview to obtain their demographic data, past medical history, smoking status, and current medications. Full blood count, renal profile, liver function test, fasting serum lipid profile, HbA1c, and urine protein creatinine index (UPCI) were collected and processed at the hospital laboratory as part of routine investigations.

### Assessment of retinal changes

All participants were sent for fundus photography and OCT at the Ophthalmology Department. Both eyes were assessed but the image of the right eye was chosen by default. If the right eye images were uninterpretable, then the left eye would be used for assessment.

#### Retinal vessel calibre (CRVE and CRAE)

**Fig. 1:**
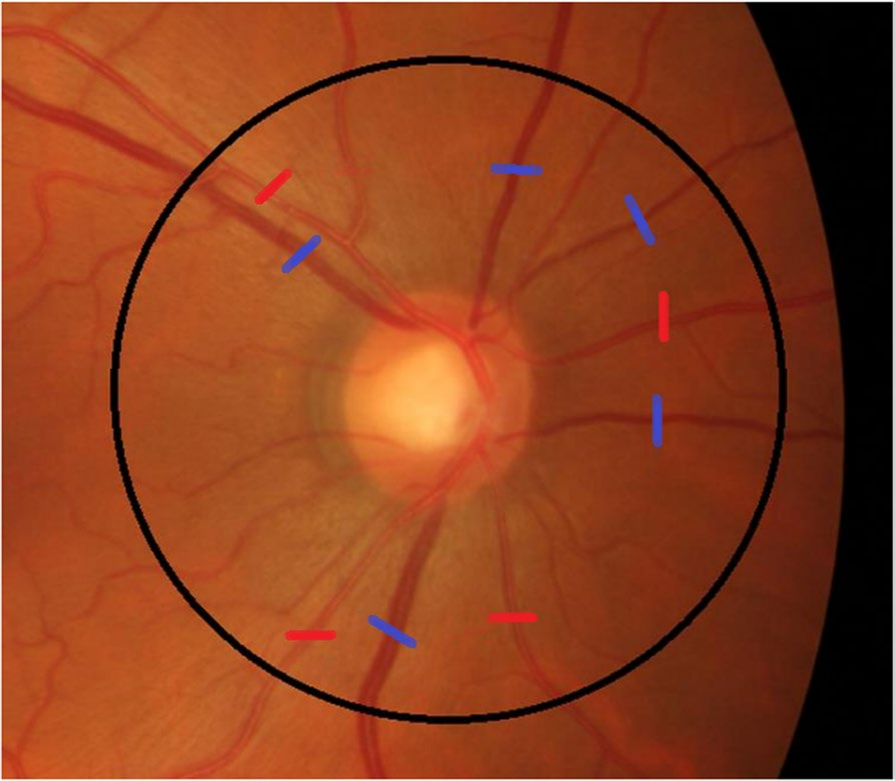
Fundus photo of a patient with the red lines representing arteries and the blue lines representing veins (published with consent)

Colour retinal photographs of both eyes were taken after dilating the pupils with 1% tropicamide and 2.5% phenylephrine hydrochloride with a digital mydriatic retinal camera (Topcon Retinal Camera TRC-50DX [type 1A], Tokyo, Japan). One retinal image of each eye was obtained to assess the retinal vessel calibre. The fundus photos were segmentalised and the four largest arteries and veins were manually selected at the region 0.5 to 1.0 disc diameters away from the disc margin (refer Fig. 1). Retinal arteriolar and venular calibres were summarised as CRAE and CRVE respectively. CRAE and CRVE were calculated by the engineering team based on the revised Knudtson-Parr-Hubbard formula [22] using an automated computer-assisted programme.

#### Retinal vessel tortuosity

The fundus photos were then sent to the Centre for Integrated Systems Engineering & Advanced Technologies (INTEGRA), Department of Electrical, Electronic and Systems Engineering, Faculty of Engineering and Built Environment, UKM for retinal vessel tortuosity assessment using the MATLAB tool. The formula used for calculating vessel tortuosity is as follows [23, 24]:

$$\begin{array}{l}Tourtousity=\frac{{d}_{curve}}{{d}_{straight}}\\ {d}_{curve}=\sum_{i=1}^{N-1}\sqrt{{\left({x}_{i+1}-{x}_{i}\right)}^{2}+{\left({y}_{i+1}-{y}_{i}\right)}^{2}},\\ {d}_{straight}=\sqrt{{\left({x}_{N}-{x}_{1}\right)}^{2}+{\left({y}_{N}-{y}_{1}\right)}^{2}}\end{array}$$

where

*(x*_*i*_*, y*_*i*_*)* = coordinates of *i*th pixel in the vessel segment

N = vessel segment constituent points

### Macula volume

**Fig. 2:**
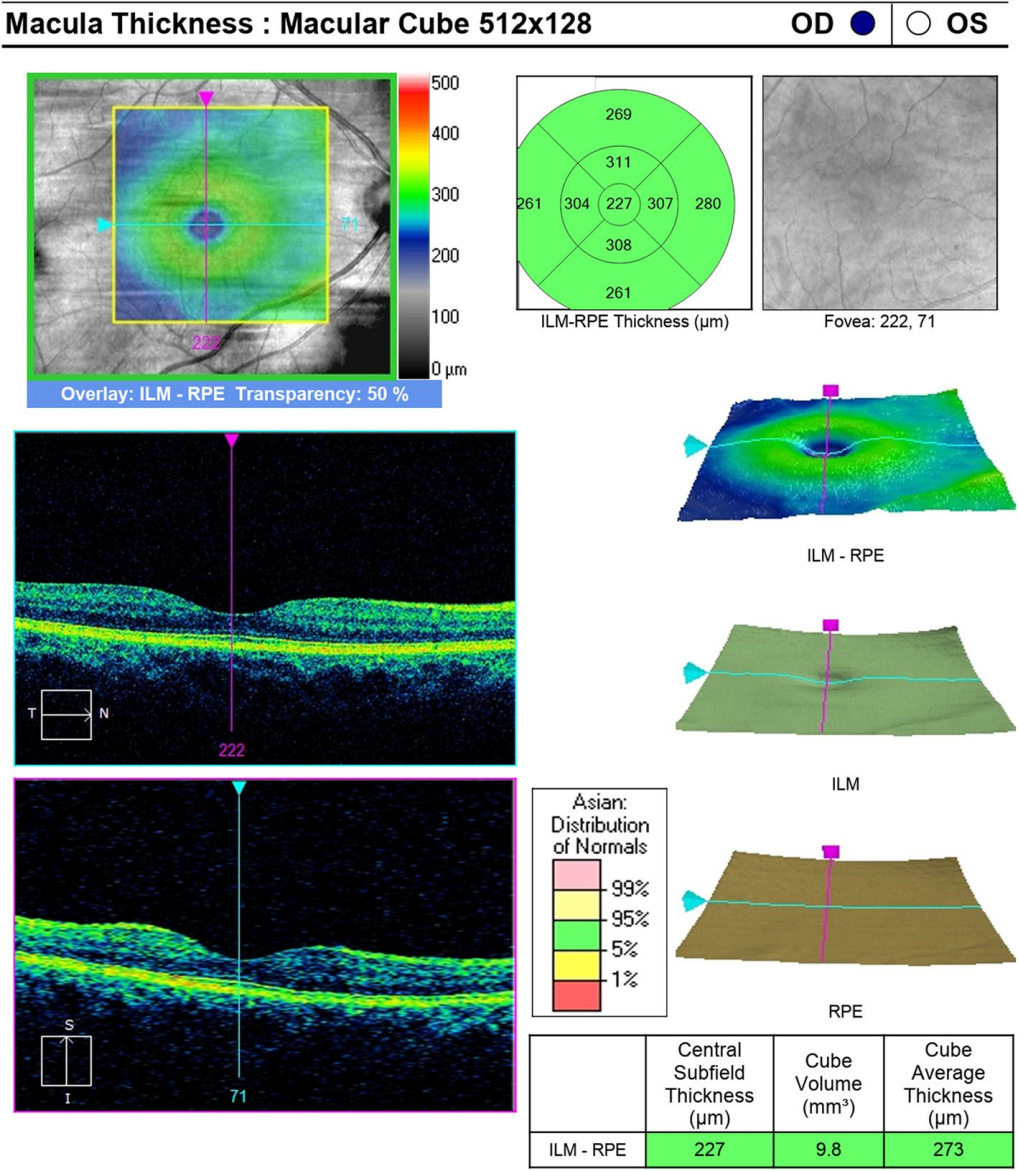
Visual of the OCT measurement of macular volume of a patient (published with consent)

Macula volume was measured via OCT using the OCT Spectralis Machine manufactured by Heidelberg Engineering, Germany. A macular scan was performed in an area of 6 × 6mm^2^ using the “Fast Macula” protocol, a standard protocol for macular cube scan. Based on the definition provided by the Early Treatment Diabetic Retinopathy Study [25], the built-in software was used to produce retinal thickness maps that were subsequently split into nine average retinal subfields with a 6 mm diameter circle centred at the true fovea location. The overall macular thickness and macular cube volume over the entire grid were derived from the output of the computer software [26]. Refer Fig. 2.

### Assessment for cardiac biomarkers

#### Hs-CRP

Blood samples for hs-CRP were sent to the Pantai Hospital laboratory and analysed using Latex Enhanced Immunoturbidimetric (Roche Diagnostic Corp) using Cobas INTEGRA Instruments. A hs-CRP level of < 1 mg/L signified low cardiovascular risk, 1–3 mg/L indicated moderate cardiovascular risk, and > 3 mg/L showed high cardiovascular risk [27].

#### ADMA

Peripheral blood taken for ADMA assessment was kept in EDTA tubes at -20ºC. It was then measured using universal ADMA ELISA kits (Novus Biologicals) by the scientific officer (single operator) in the UKM laboratory. Based on Nemeth et al. [28], a normal range was taken as 50–180 ng/ml.

### Statistical analysis

Data were analysed using IBM®SPSS software version 26. All missing data were treated by the series mean calculation method. All data were tested for normality. Normally distributed data were expressed as mean ± standard deviation (SD) whereas non-normally distributed data were expressed as median with interquartile range (IQR) (25^th^ and 75^th^ percentile). Non-parametric data were analysed using Kruskal–Wallis or Mann–Whitney U test whereas normally distributed data were analysed using t-test or ANOVA. The correlation (r) between any two parameters was analysed using Pearson test. Multiple linear regression was used for multivariable analysis to test main predictors for each of the five criteria for retinal changes (tortuosity, CRVE, CRAE and macula volume). The enter method of linear regression was used for the analysis. There was no interaction or multicollinearity between the dependent variables. A *p*-value of less than 0.05 was considered to indicate significance in all tests.

## Results

The participants’ baseline characteristics are shown in Tables 1 and 2. The 84 patients were evenly distributed with 28 patients each in CKD groups 3, 4, and 5. The distribution by gender, age, ethnicity, BMI, and blood pressure was similar between the three groups. The mean age of the participants was 58.8 ± 11.2 years. There were 44 males (52.4%) and 40 females (47.6%). Ethnically, the majority of our participants were Malays (76.2%), followed by Chinese (19.0%), and Indians (4.8%).

**Table 1:** Comparisons of demographic characteristics between participants with CKD stages 3, 4, and 5

Demographic	All (*n* = 84)	CKD stage 3 (*n* = 28)	CKD stage 4 (*n* = 28)	CKD stage 5 (*n* = 28)	*p*-value
**Gender**					
Male	44 (52.4)	17 (38.6)	14 (31.8)	13 (29.5)	0.538^a^
Female	40 (47.6)	11 (27.5)	14 (35)	15 (37.5)	
**Age**					
Years	58.8 ± 11.7	55.21 ± 10.181	57.5 ± 11.462	57.75 ± 13.525	0.381^b^
**Ethnicity**					
Malay	64 (76.2)	23 (35.9)	21 (32.8)	20 (31.3)	0.0875^a^
Chinese	16 (19.0)	4 (25)	6 (37.5)	6 (37.5)	
Indian	4 (4.8)	1 (25)	1 (25)	2 (50)	
**Cause of CKD**					
DM	43 (51.2)	14 (32.6)	11 (25.6)	18 (41.9)	0.178^a^
Hypertension	6 (7.1)	2 (33.3)	3 (50)	1 (16.7)	
IgAN	10 (11.9)	5 (50)	2 (20)	3 (30)	
FSGS	5 (6)	2 (40)	3 (60)	0 (0)	
Lupus nephritis	6 (7.1)	3 (50)	2 (33.3)	1 (16.7)	
Obstructive Uropathy	6 (7.1)	1 (16.7)	5 (83.3)	0 (0)	
Medullary Nephrocalcinosis	2 (2.4)	0 (0)	0 (0)	2 (100)	
Neurogenic Bladder	1 (1.2)	1 (100)	9 (0)	0 (0)	
VUR	1 (1.2)	0 (0)	1 (50)	0 (0)	
Single functioning Kidney post Nephrectomy	2 (2.4)	0 (0)	1 (50)	0 (0)	
ADPKD	1 (1.2)	0 (0)	0 (0)	1 (100)	
Unknown primary	1 (1.2)	0 (0)	0 (0)	1 (100)	
**Diabetes mellitus**	54 (64.3)	18 (33.3)	15 (27.8)	21 (38.9)	0.247^a^
**Hypertension**	78 (92.9)	27 (34.6)	26 (33.3)	25 (32.1)	0.584^a^
**IHD**	20 (23.8)	6 (30)	7 (35)	7 (35)	0.936^a^
**Stroke**	13 (15.5)	5 (38.5)	3 (23.1)	5 (38.5)	0.695^a^
**PVD**	4 (4.8)	0 (0)	0 (0)	4 (100.0)	**0.015**^**a**^
**Diabetic retinopathy**					
Yes	18 (21.4)	5 (27.8)	4 (22.2)	9 (50)	0.194^a^
No	36 (42.9)	13 (34.5)	11 (27.6)	12 (37.9)	
Not diabetic	30 (35.7)	10 (35.1)	13 (43.2)	7 (21.6)	
**Smoker**	5 (6)	2 (40)	1 (20)	2 (40)	0.808^a^
**Treatment**					
ACEI	25 (29.8)	11 (44.0)	11 (44.0)	3 (12.0)	0.026^a^
ARB	26 (30.9)	13 (50.0)	10 (38.5)	3 (11.5)	0.012^a^
Beta blocker	32 (38.1)	11 (34.4)	9 (28.1)	12 (37.5)	0.702^a^
CCB	57 (67.9)	18 (31.6)	17 (29.8)	22 (38.6)	0.318^a^
Statin	69 (82.1)	24 (34.8)	22 (31.9)	23 (33.3)	0.903^a^
Insulin	30 (35.7)	8 (26.7)	8 (26.7)	14 (46.7)	0.172^a^

*Abbreviations*: *Dm*diabetes mellitus, *IgAN* Immunoglobulin A nephropathy, *FSGS* Focal segmental glomerulosclerosis, *VUR* Vesicoureteral reflux, *ADPKD* Autosomal dominant polycystic kidney disease, *IHD* Ischaemic heart disease, *PVD* Peripheral vascular disease, *ACEI* Angiotensin converting enzyme inhibitors, *ARB* Angiotensin receptor blockers

^a^Chi-Square test

^b^Kruskal Wallis test

**Table 2:** Comparison of baseline clinical and laboratory characteristics between participants with CKD stage 3, 4, and 5

Clinical Parameter	All (*n* = 84)	CKD stage 3 (*n* = 28)	CKD stage 4 (*n* = 28)	CKD stage 5 (*n* = 28)	*p*-value
eGFR (ml/min/1.73m^2^)	24.0 ± 11.9	36 (33.0–38.8)	24.5 (19.0–28.0)	12.3 (8.3–14.0)	** < 0.001**^b^
Systolic BP (mmHg)	143.3 ± 19.2	146 ± 19.9	140 ± 15.7	142 ± 20.6	0.672^b^
Diastolic BP (mmHg)	78.6 ± 11.9	80 ± 13.6	80 ± 8.4	76 ± 12.0	0.119^b^
BMI (kg/m^2^)	28.8 ± 5.7	29.9 ± 5.34	27.2 ± 5.0	29.1 ± 6.6	0.143^b^
UPCI (g/mmol) (range < 0.01)	0.27 ± 0.35	0.13 ± 0.2	0.22 ± 0.2	0.46 ± 0.3	**0.001**^b^
HbA1c (%)	6.3 (5.5–7.2)	6.3 (5.5–7.1)	6.5 (5.4–7.5)	6.2 (5.5–7.2)	0.933^b^
Haemoglobin (g/dl)	12.1 (10.1–13.7)	13.5 (12.1–14.9)	12.8 (10.9–14.4)	9.8 (9.1–11.1)	** < 0.001**^b^
Hs-CRP (mg/L) (range < 1)	3.8 (0.6–11.4)	2.95 (0.5–8.08)	1.9 (0.53- 8.0)	9.85 (1.18–21.73)	**0.037**^b^
ADMA (ng/ml) (range 50–180) (28)	567.399 (410.382–710.177)	546.996 (450.619–652.805)	510.996 (374.337–658.227)	633.059 (426.677–933.278)	0.224^b^
Tortuosity	1.026 (1.021–1.030)	1.0249 (1.020–1.032)	1.0235 (1.020–1.0263)	1.0261 (1.0228–1.0372)	**0.032**^b^
CRVE (µm)	159.776 (128.500 -208.733)	159.776 (128.545–170.242)	140.651 (107.926–190.413)	200.359 (144.377–258.980)	**0.01**^b^
CRAE (µm)	151.166 (136.578–166.995)	149.798 (136.578–161.398)	150.222 (124.130–174.568)	153.819 (142.429–166.407)	0.612^b^
Macula volume (mm^3^)	9.6 (9.2–10.1)	9.75 (9.15–10.1)	9.58 (9.13–10.30)	9.58 (9.2–10.0)	0.755^b^

*Abbreviations*: *BMI* Body mass index, *SBP* Systolic BP, *DBP* Diastolic BP, *LDL* Low density lipoprotein, *HbA1c* Glycosylated haemoglobin, *UPCI* Urine protein creatinine index, *Egfr* Estimated glomerular filtration rate, *hs-CRP* High sensitivity C-reactive protein, *ADMA* Asymmetric dimethylarginine

^a^Chi Square test

^b^Kruskal Wallis test

Among the participants, more than half (*n* = 54, 64.3%) were diabetic. However, only 43 out of the 54 patients (79.6%) had CKD attributed to DM. Out of these 43 patients, 65% were males. The remaining 11 diabetic patients had CKD attributed to other causes, namely two due to obstructive uropathy, eight due to chronic glomerulonephritis, and one due to longstanding hypertension. From the results, a higher number of CKD with structural aetiologies such as obstructive uropathy, vesicoureteral reflux (VUR), and post nephrectomy were in CKD stage 4 as compared to stages 3 and 5.

In addition, all three groups of CKD had a similar distribution of DM, hypertension, ischaemic heart disease (IHD), stroke, and smoking. However, those in CKD stage 5 had a higher incidence of peripheral vascular disease (*p*-value = 0.015). There were significantly fewer patients who were on ACE-I (*p*-value = 0.012) and ARB (*p*-value = 0.012) in the CKD stage 5 group compared to stages 3 and 4 (Table 1).

Among patients with lower stages of CKD, they showed higher proteinuria levels (*p*-value = 0.001), lower haemoglobin (*p*-value < 0.001), higher hs-CRP levels (*p*-value = 0.037), more tortuous retinal vessels (*p*-value = 0.032), and higher venular calibre (*p*-value = 0.01) based on CRVE. However, there was no significant difference between the CKD groups in terms of ADMA, arterial calibre based on CRAE, and macula volume (Table 2).

### Retinal vessel tortuosity

The correlational analysis of CKD patients showed that retinal vessel tortuosity had a weak negative correlation with eGFR (*r* = -0.220, *p* = 0.044) (Table 3). However, there was no association between tortuosity and diabetes (*p* = 0.355), IHD (*p* = 0.592), or diabetic retinopathy (*p* = 0.361) (Table 4).

**Table 3:** Correlation between CKD stage (based on eGFR, creatinine, and proteinuria) with retinal changes and cardiac biomarkers

Parameters	Tortuosity		CRVE		CRAE		Macula volume		Hs-CRP		ADMA	
	**r**	***p*-value**	**r**	***p*-value**	**r**	***p*-value**	**r**	***p*-value**	**r**	***p*-value**	**r**	***p*-value**
eGFR (ml/min/1.73m^2^)	-0.220	**0.044**	-0.236	**0.031**	-0.032	0.771	0.056	0.611	-0.370	**0.001**	-0.094	0.397
Creatinine (µmol/L)	0.201	0.067	0.280	**0.01**	0.047	0.669	0.018	0.873	0.624	** < 0.001**	0.053	0.629
Proteinuria (UPCI g/mmol)	0.103	0.352	0.342	**0.001**	0.050	0.652	0.101	0.360	0.313	**0.004**	0.107	0.333

^a^Pearson test unless otherwise stated

*Abbreviations*: *r* Correlation coefficient, *eGFR* Estimated glomerular filtration rate, *CRVE* Central retinal venular equivalent, *CRAE* Central retinal arteriolar equivalent, *hs-CRP* High sensitivity C-reactive protein, *ADMA* Asymmetric dimethylarginine

**Table 4:** Association between diabetes mellitus (DM), ischaemic heart disease (IHD), and diabetic retinopathy (DR) with retinal changes and cardiac biomarkers

Association	Median (IQR)		Z-score	*p*-value	Median (IQR)		Z-score	*p*-value	Median (IQR)		Z-score	*p*-value
	**DM (*n* = 54)**	**Non-DM (*n* = 30)**			**IHD (*n* = 20)**	**Non-IHD (*n* = 64)**			**DR (*n* = 18)**	**No DR (*n* = 66)**		
Tortuosity	1.0255 (1.0210–1.0332)	1.0248 (1.0210–1.0248)	-0.924	0.355	1.025 (1.020–1.031)	1.025 (1.021–1.030)	-0.536	0.592	1.026 (1.022–1.031)	1.025 (1.021–1.030)	-0.914	0.361
CRVE (µm)	169.817 (138.927–227.931)	142.377 (113.003–166.829)	-2.334	**0.020**	155.406 (102.856–208.733)	160.778 (129.278–214.447)	-0.751	0.453	162.035 (128.500–205.348)	159.776 (128.133–205.350)	-1.444	0.149
CRAE (µm)	151.662 (142.594–165.770)	143.650 (123.011–171.060)	-1.718	0.086	157.483 (145.433–184.410)	149.798 (135.367–164.589)	-1.801	0.072	155.627 (144.184–166.737)	150.222 (132.236–168.525)	-1.433	0.152
Macula volume (mm^3^)	9.6 (9.2–10.0)	9.8 (9.3–10.3)	-1.562	0.118	9.5 (9.1–10.0)	9.6 (9.3–10.2)	-2.078	**0.038**	9.6 (9.1–10.1)	9.6 (9.2–10.1)	-0.484	0.628
hs-CRP (mg/L)	4.9 (0.7–11.7)	1.7 (0.5–8.7)	-1.684	0.092	5.2 (1.9–10.9)	3.0 (0.5–11.5)	-1.040	0.298	9.9 (0.9–21.2)	3.5 (0.6–9.4)	-1.662	0.097
ADMA (ng/ml)	592.275 (418.911–713.337)	544.244 (364.049–687.206)	-0.28	0.779	513.783 (352.555–742.937)	572.174 (428.518–694.855)	-0.730	0.465	628.132 (414.111–695.070)	552.603 (410.382–710.177)	-0.184	0.854

*Abbreviations*: *CRVE* Central retinal venular equivalent, *CRAE* Central retinal arteriolar equivalent, *IHD* Ischaemic heart disease, *IQR* Interquartile range, *hs-CRP* High sensitivity C-reactive protein, *ADMA* Asymmetric dimethylargini

^a^Mann Whitney U test unless otherwise stated

After adjustment for age, gender, BMI, smoking status, diabetes status, blood pressure, LDL, HbA1c, haemoglobin, eGFR, proteinuria, hs-CRP, and ADMA in the linear regression model, gender was found to influence retinal vessel tortuosity whereby males had more tortuous vessels by 0.008 (*p*-value = 0.031, B = -0.008). Also, for every one unit of HbA1c increment, there was a 0.003 increase in tortuosity (*p*-value = 0.047, B = 0.003) (Table 5).

**Table 5:** Predictors of retinal changes based on linear regression model

Factors	Tortuosity (*r*^2^ = 0.066)		CRVE (*r*^2^ = 0.183)		CRAE (*r*^2^ = 0.024)		Macula Volume (*r*^2^ = -0.015)	
	**Β coefficient**	***p*-value**	**B coefficient**	***p*-value**	**B coefficient**	***p*-value**	**B coefficient**	***p*-value**
**Age**	0.000	0.602	1.626	**0.010**	0.376	0.252	0.005	0.567
**Gender**	-0.008	**0.031**	-16.8	0.268	-15.6	0.053	-0.134	0.561
**BMI**	0.000	0.174	2.472	0.058	0.896	0.189	0.013	0.501
**Smoking**	0.001	0.926	16.9	0.515	-3.71	0.787	1.0	**0.012**
**Diabetes**	-0.005	0.279	-6.16	0.750	5.77	0.572	-0.615	**0.040**
**SBP**	0.000	0.067	0.071	0.861	0.195	0.364	0.009	0.153
**DBP**	0.000	0.210	-0.106	0.916	-0.442	0.267	-0.013	0.260
**LDL**	-0.001	0.586	4.442	0.510	2.371	0.505	-0.062	0.548
**HbA1c**	0.003	**0.047**	6.96	0.320	-2.055	0.578	0.098	0.357
**Hb**	-0.002	0.091	-6.47	0.121	-3.406	0.122	-0.019	0.761
**UPCI**	-0.006	0.279	36.49	0.116	2.805	0.817	0.256	0.464
**eGFR**	0.000	0.472	-0.194	0.791	0.346	0.373	0.009	0.438
**Hs-CRP**	0.000	0.253	-1.336	**0.045**	-0.598	0.088	-0.004	0.667
**ADMA**	0.000	0.279	0.026	0.183	0.006	0.553	0.001	0.086

^*^Multivariate linear regression model

*Abbreviations*: *BMI* Body mass index, *SBP* Systolic BP, *DBP* Diastolic BP, *LDL* Low density lipoprotein, *HbA1c* Glycosylated haemoglobin, *Hb* Haemoglobin, *UPCI* Urine protein creatinine index, *eGFR* Estimated glomerular filtration rate, *hs-CRP* High sensitivity C-reactive protein, *ADMA* Asymmetric dimethylarginine

### Retinal CRVE

CRVE showed a weak positive correlation with creatinine (*r* = 0.280, *p* = 0.01) and proteinuria (*r* = 0.342, *p* = 0.001) as well as a weak negative correlation with eGFR (*r* = -0.236, *p* = 0.031) (Table 3). Diabetic patients had higher CRVE values (*p*-value = 0.02)(Table 4). Age was a significant predictor of CRVE, with every increment of age in years leading to an increase in CRVE by 1.626 µm (*p*-value = 0.010, B = 1.626). In contrast, for every hs-CRP increment, there was a reduction in CRVE by 1.336 µm (*p*-value = 0.045, B = -1.336) (Table 5).

### Retinal CRAE

CRAE did not correlate with eGFR, creatinine, or proteinuria level (Table 3). There was also no association with diabetes status (*p* = 0.086), IHD (*p* = 0.072), or diabetic retinopathy (*p* = 0.152) (Table 4). No factors were found to influence CRAE based on the linear regression model (Table 5).

### Macula volume

There was no correlation between macula volume with eGFR, creatinine, or proteinuria level (Table 3). However, patients with IHD were found to have a reduced macula volume (*p* = 0.038). Based on the linear regression model, smoking and diabetic status were significant predictors of macula volume; smoking increased macula volume by 1 mm^3^ (*p*-value = 0.012, B = 1) while diabetic patients had a lower macula volume by 0.615 mm^3^ (*p*-value = 0.041, B = -0.615) (Table 5).

### Hs-CRP

Next, hs-CRP showed a weak negative correlation with eGFR (*r* = -0.370, *p* = 0.001), a moderate correlation with creatinine (*r* = 0.624, *p* =  < 0.001), and a weak positive correlation with proteinuria (*r* = 0.313, *p* = 0.004) (Table 3). However, there was no association with diabetic status (*p* = 0.092), IHD (*p* = 0.298), or diabetic retinopathy (*p* = 0.097) (Table 4).

### ADMA

ADMA levels did not have any correlation with eGFR, creatinine, or proteinuria level (Table 3). There was no association with diabetes status (*p* = 0.779), IHD (*p* = 0.465) or diabetic retinopathy (*p* = 0.854) (Table 4).

Although there were only weak to moderate correlations for eGFR, creatinine, and proteinuria with retinal tortuosity, CRVE, and hs-CRP, the post-hoc analysis showed statistically significant differences in the tortuosity, CRVE, and hs-CRP when grouped into stages i.e. between CKD stages 3 and 5 (*p*-value: CRVE = 0.015, hs-CRP = 0.032) and CKD stage 4 and 5 (*p*-value: tortuosity = 0.013, CRVE = 0.007, hs-CRP = 0.023). However, no statistically significant difference was detected in the tortuosity, CRVE and hs-CRP between CKD stages 3 and 4 (*p*-value: tortuosity = 0.128, CRVE = 0.385, hs-CRP = 0.844) (Table 6). This concurs that the most dramatic changes of retinal vessel tortuosity, CRVE and hs-CRP occur during the late stage of CKD at stage 5.

**Table 6:** Post-hoc analysis result of the association between different CKD stages with CRVE and hs-CRP

CKD stages	Stage 3 and 4 (*p*-value)	Stage 4 and 5 (*p*-value)	Stage 3 and 5 (*p*-value)
Tortuosity	0.128	**0.013**	0.184
CRVE	0.385	**0.007**	**0.015**
Hs-CRP	0.844	**0.023**	**0.032**

*Abbreviations*: *CRVE* Central retinal venular equivalent, *hs-CRP* High sensitivity C-reactive protein

^a^Mann Whitney U test unless otherwise stated

## Discussion

Among the patients with CKD stages 3 and below, diabetic kidney disease (51.2%) was the main cause of CKD. This was consistent with the reported data in the Malaysian National Renal Registry 2018 [29]. Amongst the participants with DM (64.3%), one-third of them had been diagnosed with diabetic retinopathy based on previous eye assessments. Generally, CKD in diabetic patients is usually attributed to DM unless other causes are evident [30]. In this study, 11 out of 54 (20%) of the diabetic patients also had other underlying significant structural abnormalities that caused CKD as shown by their kidney biopsy findings or imaging showing.

Known risk factors for CVD include obesity, smoking, dyslipidaemia, hypertension, DM, family history of premature coronary disease, CKD, and albuminuria [31]. CKD, especially among those with DM, predisposes to a high risk of atherosclerotic CVD (ASCVD), including coronary artery disease, peripheral vascular disease, and stroke. From our observation, 23.8% of our patients had IHD, 15.5% had a stroke before, and 4% had peripheral vascular disease. Only 6% of patients included in the study were smokers, which was low compared to the prevalence of smokers among the general population of Malaysia.

The comparison between patients in CKD stages 3, 4, and 5 showed a significant difference in eGFR, proteinuria level, haemoglobin level, hs-CRP, retinal vessel tortuosity, and CRVE results, as well as the use of ACE-I and ARBs between the groups. For instance, higher proteinuria was found in those with a lower stage of CKD, likely due to the degree of renal injury in these patients. However, it can also be attributable to the low usage of antiproteinuric agents such as ACE-I or ARB due to the risk of hyperkalaemia and reduced eGFR in this group of patients.

Subsequent correlational analysis between retinal changes and CKD stages showed that a lower eGFR, higher creatine level, and proteinuria (all signs of lower stage of CKD) were correlated with a higher CRVE, even though it was a weak correlation. CRVE was also significantly correlated with diabetes status, whereby diabetics were found to have a higher CRVE compared to non-diabetics. These findings concurred with the results from Yip et al. [5] and Liew et al. [2]. Similarly, the Wisconsin Epidemiologic Study of Diabetic Retinopathy (WESDR) [32] also highlighted an association between CKD and a higher venular calibre, especially among diabetics.

In addition, patients with lower eGFR were found to have more tortuous retinal vessels. It was proven by Sasongko, MB et al. in which a greater retinal vessel tortuosity was independently associated with retinopathy and early-stage nephropathy in type 1 diabetes [10]. Tortuosity and venular calibre are structural changes of the retina that happen as a result of endothelial dysfunction and haemodynamic instability in CKD patients [10, 33, 34]. This is likely attributed to the increased inflammatory states in CKD which leads to increased permeability of the vessels and thickening of the basement membrane, which in turn increases the venular calibre [33]. The pathophysiology behind retinal vessel tortuosity is postulated to be due to the constant inflammatory state in CKD leading to dysregulation of blood flow [35, 36], tissue hypoxia, endothelial dysfunction [37], and increased levels of vascular endothelial growth factor (VEGF) [38] causing vessel wall weaking and fragility, and consequently remodelling occurs.

In our study population, no correlation was detected between CKD stages with CRAE, possibly due to the small sample size and the difference in the genetic and environmental profile of our patients compared to previous studies. Asians have a different profile of contributing factors to CKD compared to Caucasians. In the literature, two studies by Sabanayagam et al. [7, 8] among Singaporean patients showed an association between retinal arteriolar narrowing and CKD. Even though the two countries were geographically near, the Singaporean study had a bigger sample size but only comprised patients of Chinese ethnicity, as compared to the multi-ethnic patient population in our study.

Furthermore, in our study, patients with IHD had a lower macula volume. A study by Balmforth et al. [9] found that patients with CKD had reduced macula volume and that reduced macula volume was also associated with higher levels of systemic inflammation, as measured by hs-CRP. The elevated level of this inflammatory marker could be representative of a generalised systemic microvascular injury confined not only to the kidneys, but also involving the cardiovascular system. Unfortunately, this was not reflected in our study as no association was found between hs-CRP and IHD, despite hs-CRP being one of the most sensitive markers of inflammation [39]. Macula volume is thought to be affected due to the atrophy of the cells as a result of compromised blood supply caused by microvascular injury [9].

On the other hand, proteinuria was associated with a higher hs-CRP level in our study population. With a higher level of proteinuria as CKD progresses, hs-CRP clearance is likely reduced in these patients, thus resulting in higher hs-CRP levels in the body [40]. It is known that hs-CRP can reduce the expression of nitric oxide synthase, leading to inflammation and subsequent development of atherosclerosis. As a result, the CKD patients remain in a state of chronic low-grade inflammation as observed by Abraham et al. [11]. In short, these findings support the fact that proteinuria is a known risk factor for ASCVD [31].

In contrast to previous studies supporting ADMA association with reduced macula volume and IHD [9, 13], ADMA levels did not show a significant association with any parameters in this study, possibly due to the fact that the majority of our patients did not have IHD. However, ADMA level was elevated in our CKD patients compared to the normal population based on the reference levels in Nemeth et al. [28], thus proving that even CKD stage 3 is a definite risk factor for ASCVD.

Next, no significant association was detected between diabetic retinopathy with retinal vascular and macula changes, although Sasongko et al. [41]. previously demonstrated an increased retinal vessel tortuosity in association with diabetic retinopathy. This could be due to the fact that only one-fifth (21%) of patients in this study were diagnosed with diabetic retinopathy. A significant number of patients with diabetic retinopathy had previously undergone photocoagulation retinal therapy and were excluded from this study as their retinal vessels could not be accurately assessed.

Last but not least, the linear regression analysis found that CKD patients of older age had poorer CRVE outcomes, independent of other factors. This could be explained by older patients having a longer duration of CKD. Furthermore, CKD deteriorates further with age and the prolonged proinflammatory processes on the vessel can lead to further weakening and dilatation. On the other hand, macula volume was affected by smoking status and diabetic status after adjusting for other factors whereby a higher macula volume was found in smokers and non-diabetics. This was in contrast with previous studies that showed smoking either reduced [42] or had no effect [43] on macula volume. Reduced macula volume is thought to be a consequence of the vasoactive effect of smoking and the oxidative stress that produce hypoxia and ischaemia [42], similar to how retinal blood flow becomes impaired in patients with diabetes [44]. Interestingly, the male gender was found to have an increased vessel tortuosity based on this model, a new finding that was not reported elsewhere previously, possibly due to the fact that the majority of patients with CKD due to diabetes in this study were male patients (65%). In a post-hoc analysis, diabetics are found to have more tortuous vessels. The increment in Hba1c also contributed to increased vessel tortuosity as previously reported [41]. Increment in hs-CRP also independently caused an increase in CRVE, likely attributable to an inflammatory state that leads to endothelin dysfunction and increased vessel permeability [33, 34].

One of the main limitations in our study was the convenience sampling that might have contributed to selection bias. In addition, although the storage and testing for ADMA were done manually by a single operator in a consistent manner, it was however performed in two batches, thereby creating potential bias in testing. Additionally, future studies should consider the use of prospective design with the inclusion of a larger number of participants at earlier stages of CKD. This will provide more information to ascertain the degree and the starting point of retinal changes and cardiac biomarkers in CKD.

## Conclusion

In conclusion, our study showed that eGFR value affects retinal vessel tortuosity, CRVE and hs-CRP. These parameters bear potential to be used as non-invasive tools in assessing CKD. However, only macula volume may be associated with CVD risk among the CKD population.

## Data Availability

The datasets used and analysed are available from the corresponding author on reasonable request.

## Abbreviations

ACEI: Angiotensin converting enzyme inhibitor
ADMA: Asymmetric dimethylarginine
ADPKD: Autosomal dominant polycystic kidney disease
ARB: Angiotensin receptor blocker
ASCVD: Atherosclerotic cardiovascular disease
BMI: Body mass index
BP: Blood pressure
CCB: Calcium channel blocker
CKD: Chronic kidney disease
CRAE: Central retinal arteriolar equivalent
CRVE: Central retinal venular equivalent
CVD: Cardiovascular disease
DM: Diabetes mellitus
eGFR: Estimated glomerular filtration rate
ELISA: Enzyme linked immunosorbent assay
ESRD: End stage renal disease
FSGS: Focal segmental glomerulosclerosis
hbA1c: Glycosylated haemoglobin
Hs-CRP: High sensitivity C-reactive protein
IgAN: Immunoglobulin A nephropathy
IHD: Ischaemic heart disease
LDL: Low density lipoprotein
MDRD: Modification of diet in renal disease
OCT: Optical coherence tomography
PVD: Peripheral vascular disease
UPCI: Urine protein creatinine index
VUR: Vesicouereteric reflux
